# Digital radiography reject analysis: A comparison between two radiology departments in New Zealand

**DOI:** 10.1002/jmrs.654

**Published:** 2023-01-19

**Authors:** Gabriela Bantas, Rhonda‐Joy Sweeney, Sibusiso Mdletshe

**Affiliations:** ^1^ Department of Anatomy and Medical Imaging, School of Medical and Health Sciences The University of Auckland Auckland New Zealand

**Keywords:** direct digital radiography, image reject analysis, image rejection rate

## Abstract

**Introduction:**

Image reject analysis (RA) in direct digital radiography (DDR) is an important quality indicator tool. Analysis of rejected images is a component of quality assurance (QA) programmes, with the overall aim of reducing patient radiation dose. This study aimed to compare differences in image rejection rates (RR) and the reasons for rejection between two radiology departments.

**Methods:**

A retrospective quantitative descriptive study of images performed across the two radiology departments (RAD 1 and RAD 2) acquired with DDR systems between the beginning of February and the end of May 2021 was undertaken. Collected data included the medical imaging technologist (MIT) selection of image rejection reasons for different anatomic regions and compared between the two radiology departments.

**Results:**

A total of 47,046 images and 29,279 images were acquired at RAD 1 and RAD 2, respectively, with an overall image rejection rate of 7.86% at RAD 1 and 5.91% at RAD 2. The primary reason for image rejections was positioning errors, 79.4% and 77.3% recorded at RAD 1 and RAD 2, respectively. Significant differences were demonstrated between the two radiology departments for image rejection rates and selected reasons for rejection for most anatomical body groups.

**Conclusion:**

The implementation of image RA remains a key part of QA in radiology departments utilising DDR systems. This study recommends interventions based on image RRs for examinations taking into consideration the department‐specific variations and imaging protocols used.

## Graphical abstract

Image reject analysis (RA) in direct digital radiography (DDR) is an important quality indicator tool. A retrospective quantitative descriptive study of images performed across the two radiology departments (RAD 1 and RAD 2) acquired with DDR systems between the beginning of February and to the end of May 2021 was undertaken. The findings in this study demonstrated that positioning errors attributed to MIT‐technique remain a key factor to consider when implementing QA in digital radiography image RA.

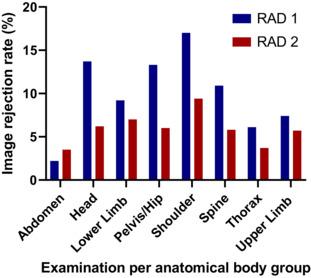

## Introduction

Image reject analysis (RA) is a quality control (QC) process and an important component of quality assurance (QA) programmes to measure the efficiency of radiology departments by determining the rates and reasons for rejected images.^1^, ^2^ A rejected image is an image that is deemed to not add any additional diagnostic value to the clinical analysis^3^ which in practice is based on the subjective evaluation by a medical imaging technologist (MIT) at the time of image acquisition.^4^ RA is a quality indicator tool for continuous quality improvement (CQI) in the planning of interventions such as MIT educational strategies, evaluating current and developing new examination protocols, assessing the level of image quality and keeping track of reducing unnecessary radiation dose to the patient.^3^, ^5^, ^6^

RA with most direct digital radiography (DDR) and computed radiography (CR) systems is now a simple and efficient process that utilises automated reject data collection and analysis software. However, this process is dependent on the subjective image quality evaluation by the MIT which can be attributed to variation in MIT clinical experience, department workflow and interpretation of the criteria for the various image rejection reasons that are displayed on the screen.^4^, ^5^

Previous studies in screen‐film radiography indicate that rejected images occurred in 6–15% of examinations.^2^ Image acquisition with CR systems has been shown to reduce image rejection rates (RR) by between 4% and 5%.^3^ International studies using DDR place image RRs between 9% and 17%, higher than those reported for CR rates.^4^, ^5^, ^7^ Image digitisation significantly changed the reasons for image rejection. Image rejections for the screen‐film systems were mostly exposure‐related.^2^ Almost all relevant studies pertaining to DDR found rejections associated with positioning of the patient to be the primary reason, followed by anatomy cut‐off.^4^, ^5^, ^7^, ^8^, ^9^ Waaler and Hofmann^2^ suggest that the probability of positioning errors would increase for larger anatomic body parts such as the abdomen and pelvic examinations. Since then, it has been shown that these anatomical regions have some of the highest image RRs with the addition of knee examinations.^4^, ^5^, ^7^

In 2015, the American Association of Physicists in Medicine (AAPM) Task Group 151 (TG151) published a report on quality control (QC) procedures, recommending an 8% image RR for DDR systems, with a ±2% variation threshold where the opportunity for possible corrective action can be implemented.^10^ However, this recommendation appears to not be internationally accepted within QA guidelines as several studies have argued that department‐specific factors relating to differences in MIT technique, working conditions and radiographic equipment will influence the department's image RRs.^4^, ^7^, ^8^ The AAPM TG151^10^ reported up to threefold variation in the percentage of image RRs among different radiology departments. Despite this variability in image RRs, the overarching purpose of image RA is to investigate and amend department‐specific issues as part of quality improvement in accordance with the as low as reasonably achievable (ALARA) principle.^11^

There is a paucity of recently published articles that are specific to DDR image reject rates that have utilised a large sample size and there have been no such studies conducted in the New Zealand setting. Thus, image RA is still viable, especially in the modern DDR era. The aim of this study was to compare the difference in image RRs and reasons for image rejections between two radiology departments using DDR systems in a large metropolitan area.

## Materials and Methods

This retrospective quantitative descriptive study was conducted in radiology departments equipped with DDR systems manufactured by Philips Healthcare at two nominated hospitals (RAD 1 and RAD 2) in New Zealand. The data were exported from the Philips Healthcare DigitalDiagnost QA software tool integrated within each DDR system at RAD 1 and RAD 2, across a 4‐month period from 1 February 2021 to 31 May 2021. Approval was given by the Auckland Health Research Ethics Committee (Reference Number 3332). Patient confidentiality was maintained throughout the study since no patient‐identifying information was collected. The Clinical Director of the two radiology departments involved in the study approved data extraction to be undertaken. Access to images and systems was supervised by the designated QA MITs.

RAD 1 included the emergency department (ED) and general radiology departments that provide medical imaging services for adult inpatients and ED patients, following acute trauma, and RAD 2 provides medical imaging services for referred adult outpatients. All X‐ray rooms at RAD 1 had the Philips DDR systems installed in 2016 with upright, table and free detectors and with the fourth version of the Philips Healthcare DigitalDiagnost QA software tool. At RAD 2, the Philips DDR systems were installed at separate time intervals in each room, which resulted in different installed versions of the Philips Healthcare DigitalDiagnost QA software tool.

The inclusion criteria were as follows:

- All acquired images (both accepted and rejected) between 1 February and 31 May 2021 from the DDR systems in rooms 1, 2, 5, 6 and 8 at RAD 2 and from rooms 1, 2, 3, 5 and 6 at RAD 1.
- Images could not be rejected without selecting a rejection reason from the drop‐down list options.

The exclusion criteria used in this study were:

- Teaching and QA service images acquired with phantoms, as they do not represent ionising radiation to patients.
- Rejected images with the rejection reason being ‘other’. This reason for image rejection cannot be tracked to an individual rejection reason and did not provide any information for future intervention purposes.
- Images that were acquired under the incorrect examination view.

Data were collected by the designated QA MIT at RAD 2 and the clinical lead MITs at RAD 1. Multiple data extraction periods were required to successfully export the data in line with the availability of the DDR rooms outside of clinic hours. Data collection was performed outside of outpatient clinic hours at RAD 2 and when time permitted at RAD 1. The data were exported from all systems and the files were saved on the QC computers as Comma‐Separated Values (CSV) files. The data were then distributed to the principal investigator after which the data from all rooms were combined on a Microsoft Excel® spreadsheet.

Initial data included the specific projection view; a subclassification of the anatomic regions that were examined. The examinations were categorised by anatomical body groups to include the abdomen, head, lower limb, pelvis/hip, shoulder, spine, thorax and upper limb, comparable to previous research methods.^5^, ^8^ This type of classification allowed enough data to be collected under each examination type.

To accommodate the initial data to the main objectives of the study, adjustments resulted following the principal investigator's interrogation of the data and the final data recorded the anatomic body part, whether the image was accepted or rejected and the reason for image rejection. Reasons for image rejection were categorised as MIT‐related and patient‐related errors as shown in Table 1.

**Table 1:** Image rejection reasons in relation to MIT‐related and patient‐related reasons.

Image rejection reason	Included or excluded in study sample
Additional Nasogastric (NG) tube manipulation	Included as MIT‐related error
Automatic exposure control (AEC) failure	Included as MIT‐related error
Collimation error/Collimation Error	Included as Collimation error as MIT‐related error
Gridlines	Included as Image artefacts as MIT‐related errors
Image artefact/Gridlines	
Image artefacts	
Other	Excluded
Patient moved	Included as patient‐related error
Poor inspiration	Included as patient‐related error
Positioning error	Included as MIT‐related error
Service testing	Excluded
Service testing/QA	Excluded
Stitching re‐adjustment	Included as MIT‐related error
Teaching images	Excluded
Teaching purposes	Excluded
Technical problem	Included as MIT‐related error
Wrong detector selected	Included as MIT‐related error
Wrong exposure	Included as MIT‐related error
Wrong projection	Included as MIT‐related error

MIT = Medical imaging Technologist; QA = Quality Assurance.

The raw data were analysed using both descriptive and inferential statistics with the assistance of a Biostatistician. The overall RR was calculated using the following formula.

$$\text{Image}\;\mathrm{RR}=\frac{\text{number of rejected images}}{\text{total number of acquired images}\;}\times 100$$

By filtering the data in Microsoft Excel, frequency distribution tables were created to calculate the number of image RRs per anatomic body part and compare between the two radiology departments. The body parts with the highest image RRs were expressed as percentages. This process was repeated for each specific radiographic projection and the reasons for image RRs. All percentages were rounded to one decimal place.

The type II Wald chi‐square goodness‐of‐fit test was used to compare the overall image RRs from the two radiology departments by comparing the differences between distribution patterns.^12^ The null hypothesis assumed that no differences existed in the overall image RRs between the two radiology departments. A *P*‐value of 0.05 or less indicated whether any significant statistical differences exist between the two variables and whether the null hypothesis is accepted or rejected.^13^

The t‐test was used to compare the means of two samples and examine the matched measurements of a group.^13^ The *P*‐value and 95% CI results were used to accept or reject the null hypothesis, which assumed no differences between the samples.^13^ The Holm correction reduced the inflation for type I errors and increased the power of the statistical test when performing multiple tests.^14^ The statistician integrated the Alpha 0.05 method and expected that one in 20 comparisons to be of significant difference.

## Results

### Comparison of overall image rejection rates

A total of 76,325 acquired images met the inclusion criteria, data from the 47,046 examinations from RAD 1 and 29,279 RAD 2 examinations were combined. A total of 3647 images were rejected in the radiology rooms at RAD 1 and 1774 images were rejected at RAD 2. This resulted in an overall image rejection rate of 7.86% (95% CI 6.85, 9) at RAD 1 and 5.91% (95% CI 5.13, 6.8) at RAD 2. A significant difference (*P* < 0.5) in the overall image RRs has been found between the two radiology departments, with Table 2 displaying these figures.

**Table 2:** Image rejection rate difference between the radiology departments at RAD 1 and RAD 2.

Hospital	Total acquired images	Rejected images (*n*)	% Of total images (95% CI)	*P*‐value
RAD 1	47,046	3647	7.86 (6.85, 9)	0.004229
RAD 2	29,279	1774	5.91 (5.13, 6.8)	

RAD = Radiology department; *n* = number; CI = Confidence Interval.

### Comparison between examination‐specific image rejection rates

**Figure 1:**
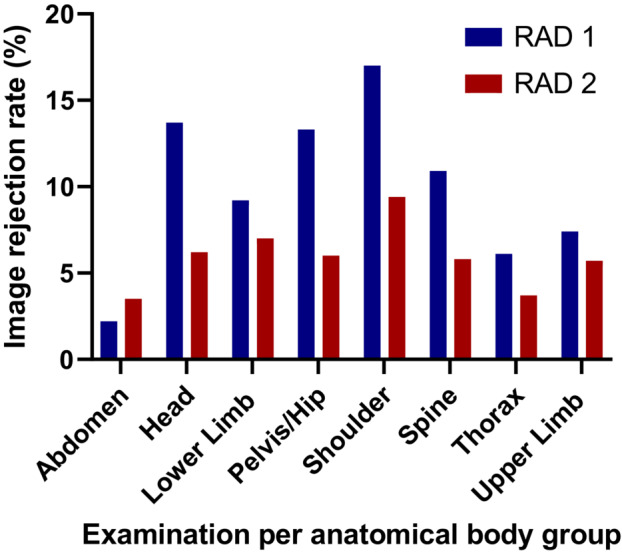
Image rejection rates per anatomical body group at the radiology departments at RAD 1 and RAD 2 hospitals during a 4‐month period.

Figure 1 demonstrates the examinations with the highest image RRs recorded at each of the radiology departments. Table 3 demonstrates the image RRs for the different examinations between the two radiology departments. The greatest difference in images rejected due to MIT‐related errors was observed in the thorax group, 3.5 times greater the RRs at RAD 1 compared to RAD 2. There was also a significant difference between the mean RRs for thorax groups due to positioning errors (*P* < 0.001), as RAD 1 recorded over double the RRs for thorax images compared to RAD 2. The pelvis/hip group recorded the greatest difference between mean RRs due to positioning errors and this was found to be significantly different (*P* < 0.001). The image RRs in the shoulder group due to positioning errors were found to be significantly different, with RAD 1 recording 13.2% (95% CI 12, 14.6) compared to 7.7 (95% CI 6.6, 8.8). The lower limb group recorded a significant difference between mean RRs due to positioning errors (*P* < 0.001). Rejected images due to positioning errors for the spine group were nearly double at RAD 1 compared to RAD 2 and the mean between RRs was found to be significantly different (*P* < 0.001).

**Table 3:** *T*‐test with Holm‐corrected *P*‐values to show difference in image rejection rates for the different examinations between the radiology departments at RAD 1 and RAD 2.

Examination	Reasons for image rejections between RAD 1 and RAD 2									
	Hospital	MIT‐related errors			Positioning errors			Patient‐related errors		
		*n*	% (CI)	*P*‐value	*n*	% (CI)	*P*‐value	*n*	% (CI)	*P*‐value
Abdomen	RAD 1	20	0.5 (0.3, 0.8)	1.00	63	1.7 (1.3, 2.1)	0.817	1	0 (0, 0.2)	0.304
	RAD 2	1	0.2 (0, 1.5)		13	2.9 (1.7, 4.9)		2	0.4 (0.1, 1.7)	
Head	RAD 1	1	1 (0.1, 6.6)	1.00	12	11.7 (6.7, 19.4)	1.00	1	1 (0.1, 6.6)	1.00
	RAD 2	1	3.1 (0.4, 19.1)		1	3.1 (0.4, 19.1)		0	0 (0, 100)	
Lower Limb	RAD 1	47	0.5 (0.4, 0.7)	1.00	741	8.3 (7.8, 8.9)	0.000	38	0.4 (0.3, 0.6)	1.00
	RAD 2	71	0.7 (0.5, 0.8)		623	5.8 (5.3, 6.2)		56	0.5 (0.4, 0.7)	
Pelvis/Hip	RAD 1	61	3.6 (2.8, 4.5)	0.00	157	9.1 (7.9, 10.6)	0.0001	10	0.6 (0.3, 1.1)	0.181
	RAD 2	12	0.5 (0.3, 0.9)		130	5.5 (4.6, 6.5)		1	0 (0, 0.3)	
Shoulder	RAD 1	80	3.2 (2.6, 3.9)	0.00	334	13.2 (12, 14.6)	0.000	16	0.6 (0.4, 1)	0.687
	RAD 2	12	0.5 (0.3, 0.9)		172	7.7 (6.6, 8.8)		26	1.2 (0.8, 1.7)	
Spine	RAD 1	106	2.8 (2.3, 3.3)	0.00	274	7.1 (6.4, 8)	0.000	39	1 (0.7, 1.4)	1.00
	RAD 2	44	0.9 (0.7, 1.2)		180	3.7 (3.2, 4.2)		59	1.2 (0.9, 1.5)	
Thorax	RAD 1	144	0.7 (0.6, 0.8)	0.001	1003	4.7 (4.4, 5)	0.000	145	0.7 (0.6, 0.8)	0.00
	RAD 2	10	0.2 (0.1, 0.3)		116	2.1 (1.8, 2.5)		76	1.4 (1.1, 1.7)	
Upper Limb	RAD 1	23	0.5 (0.3, 0.7)	1.00	312	6.5 (5.8, 7.2)	0.360	19	0.4 (0.3, 0.6)	0.360
	RAD 2	9	0.3 (0.2, 0.6)		136	4.6 (3.9, 5.4)		23	0.8 (0.5, 1.2)	

RAD = Radiology department; *n* = number; CI = Confidence Interval.

### Comparison between image rejection reasons

MIT‐related errors are classified by the technical ability of the MIT performing the radiographic examination. Patient‐related errors are attributed to patient breathing or movement which influenced the image quality outcome as assessed by the MIT at the time of acquisition. There were nine reasons for image rejection attributed to MIT‐related errors. Overall, positioning errors accounted for 92.72% and 86.3% of rejected images at RAD 1 and RAD 2 respectively. The second most common reason for image rejection at RAD 1 and RAD 2 due to MIT‐related errors was wrong exposure, 5.0% and 4.1%, respectively, as demonstrated in Table 4. There were two reasons attributed to patient‐related errors. The most common patient‐related error resulting in image rejection was patient movement, which was almost twice as high at RAD 2 (10.5%) compared to RAD 1 (5.8%). At least 50% of the image rejection reasons per anatomical body group were attributed to positioning errors at both the radiology departments, as demonstrated in Table 3.

**Table 4:** Summary of selected image rejection reasons at the radiology departments at RAD 1 and RAD 2 during a 4‐month period.

Reason for image rejection		Hospital‐specific rejected images (*n* (%))	
		RAD 1	RAD 2
MIT‐related errors	Additional NG tube manipulation	4 (0.1)	0 (0.0)
	AEC failure	46 (1.26)	0 (0.0)
	Collimation error	171 (4.69)	0 (0.0)
	Image artefacts	21 (0.575)	52 (2.9)
	Positioning error	2896 (79.4)	1371 (77.3)
	Stitching re‐adjustment	0 (0.0)	32 (1.8)
	Wrong detector selected	56 (1.5)	0 (0.0)
	Wrong exposure	183 (5.0)	72 (4.1)
	Wrong projection	1 (0.03)	4 (0.23)
Patient‐related errors	Patient moved	212 (5.8)	187 (10.5)
	Poor inspiration	57 (1.6)	56 (3.2)
Total rejected images (*n* (%))		3647 (100)	1774 (100)

RAD = Radiology department; *n* = number.

## Discussion

Acquired images which constituted both accepted and rejected images were categorised by anatomic body part as well as by the image rejection reason. The image RRs were used in line with the reasons for image rejection for the anatomic body regions to highlight areas of clinical concern. Although the overall acceptance rates at both hospitals were over 80% for all anatomic body regions, it was identified that all anatomic body regions yielded image RRs of at least 50% due to positioning errors. Positioning errors accounted for over three‐quarters of rejected images and match those rates observed in earlier studies.^4^, ^7^, ^8^

The production of high‐quality images requires MITs to guide the patient during the examination process, correctly position and select optimal exposure factors.^15^ Some authors have driven the development of categorising imaging errors as MIT‐related errors, as the technical skill influences their ability to instruct and position patients can be attributed to the clinical experience and skills of the MITs.^16^, ^17^ Patient‐related factors such as clinical presentation, mobility and age have been noted to impact image quality and included when addressing image RRs in some studies.^9^, ^18^

In the study, the most frequent image rejection reasons after positioning errors were the same for both RAD 1 and RAD 2. At RAD 1, image rejection reasons due to MIT‐related reasons included incorrect exposure (5.0%) and collimation errors (4.69%). MIT‐related errors were also the most common reasons for image rejection at RAD 2 including incorrect exposure (4.1%) and image artefacts (2.9%). An important observation should be made that collimation errors were included in the collected data from RAD 1; however, collimation errors were not selected at RAD 2. An important factor in collimation errors is X‐ray field misalignment.^19^ A source of uncertainty is the variation in MIT interpretation of image quality, and it is important to bear in mind the possible bias in the selection of image rejection reasons without a standardised method to define image rejection reasons.^6^ With the introduction of digital systems, professional interactions between MITs and radiologists have reduced as images are sent electronically.^20^, ^21^ It has been suggested that this reduction in communication regarding image quality may contribute to increasing positioning errors.^22^

In our study, exposure factors were found to be within the top three reasons for image rejection in both radiology departments. Incorrect exposure selection has been classified as a MIT‐related error; however, it is important to note that incorrect exposure settings may result in the suboptimal quality of images even with proper exposure settings by the MIT.^2^ This study did not assess the type of wrong exposure selected, but the findings by Atkinson et al.^4^ and Stephenson‐Smith et al.^7^ may help us to understand that more images are likely to be rejected due to under‐exposure as post‐processing algorithms of DDR systems are able to produce over‐exposed images of adequate image quality.

Consistent with previous publications, the thorax group was the most frequent type of examination performed at RAD 1.^6^, ^8^, ^9^ Shoulder examinations recorded the highest image RRs at both radiology departments, 13.2% at RAD 1 and 7.7% at RAD 2. This study found a significant difference (*p <* 0.05) between image RRs at RAD 1 and RAD 2 for shoulder, pelvis/hip and spine examinations due to positioning errors and other MIT‐related errors. These findings were aligned with previous reports which have shown that shoulder, knee and pelvis/hip examinations have the highest image RRs with the main reason for image rejection attributed to positioning errors.^4^, ^5^, ^7^ It is surprising to observe that Waaler and Hofmann's^2^ study is still pertinent as to which anatomic body region is expected to lead to the highest image rejection rates.

This was a robust image RA with a large data set of 76,325 acquired images, resulting in an overall image RR of 7.86% at RAD 1 which was demonstrated to be significantly higher than the overall rate of 5.91% recorded at RAD 2. The image RR recorded at RAD 1 is a more accurate representation of image RRs for emergency and inpatient examinations to the exclusion of outpatient examinations. Previous literature^4^, ^8^ reported that the image RR is expected to be higher for emergency and inpatient radiographic examinations compared to outpatient examinations and this was a noted finding in this study. However, RAD 2 (which only had outpatient examinations) recorded image RRs due to patient‐related errors that were double the rates at RAD 1. This was the only image rejection reason to have higher image RRs at RAD 2 than at RAD 1. This finding is contrary to previous studies which have suggested that inpatient examinations are more likely to result in increased rejection of images.^4^, ^8^

A source of uncertainty is the variation in MIT interpretation of image quality, and it is important to bear in mind the potential selection bias of image rejection reasons without a standardised method to define image rejection reasons.^6^ Examinations may be categorised based on anatomic regions; however, attention should be brought to the decision‐making process and selection of image rejection reason by MITs, particularly during a difficult patient presentation when detector, tube alignment and exposures have to be adapted. An important observation should be made that positioning errors and collimation errors were included in the collected data from RAD 1; however, collimation errors were not selected at RAD 2 due to non‐inclusion in the image rejection reason list. Previously, indications have been that image quality perception and subjective understanding of the definition for the image rejection reason are dependent on the clinical experience and knowledge of the MIT.^5^, ^8^ RA studies provide the opportunity to implement continuous quality improvement to monitor the impact of education and training of staff to further improve the quality of practice is a vital step to dose optimisation.^23^

### Limitations of study

This study encountered some limitations worth considering. Firstly, the data were collected during a period that included lockdown restrictions due to the COVID‐19 pandemic, which may have impacted the normal workflow of the radiology departments. It is possible that the lockdown periods due to the COVID‐19 pandemic may have influenced the duration of examinations and the technique used by the MITs, which could have impacted the rate of positioning errors.^24^, ^25^

Additionally, this study was conducted between radiology departments that had different patient populations; RAD 1 provided radiology services to inpatient and trauma patients while RAD 2 provided outpatient examinations. A repeat of this image RA at the individual radiology departments following interventions would allow for the comparison of image RRs and selection of image rejection reasons that reflect the specific department variations.

This study has found that different versions of the image RA software installed in the different DDR systems may have resulted in different image rejection reasons. A lack of standardised image rejection reasons on the drop‐down menus may impose a limiting factor when MITs evaluate the images prior to rejection. Another limitation was that the installation periods for the DDR systems were staggered with different versions of the image reject software being integrated into each system. For some X‐ray rooms, this limited the ability to adapt the image rejection reasons in the drop‐down menu list and this may have impacted the difference in image rejection reasons selected at the two radiology departments. Due to the exclusion of rejected images labelled as ‘other,’ this presents questionable evaluation by MITs and underestimates the actual image RR. Moreover, it cannot be excluded that MITs incorrectly categorised images when they were making the subjective evaluation to reject; however, the reliability of image categorisation could not be assessed, as the software does not store the rejected images.

Lastly, the findings in this study may be limited by the variations in the reject analysis software and the differences in image rejection reasons included in the drop‐down menus which may have influenced the selection of image rejection reasons.^6^, ^8^

### Recommendations for future research

There remains an opportunity for progress in determining image RRs of projections as this could identify specific areas for each examination type, allowing department‐specific analysis of existing image rejection analysis and rejection rates. In addition, future research should address the inter‐variability between MIT perceptions of image quality and assess individual criteria for image quality. Future research should also consider image RRs as part of the bi‐cycle clinical audit of the radiology department to measure whether the improvement strategies implemented have been successful in reducing image RRs. This introduces the potential for training with an emphasis on introducing examination‐specific interventions to reduce image RRs.

### Recommendations for practice

It is essential that the equipment used has standardised rejection menu logs and terminology, especially where the equipment supplier is the same, to assist with future research and training of staff. Each of the study sites needs to enhance its cyclic QA and quality control programs as a mechanism to monitor and minimise RRs. The results highlighted that both study sites have positioning errors as the primary rejection reason followed by wrong exposure factors with regard to MIT‐related errors. We, therefore, recommend that each site implements a strategy to reduce positioning‐related rejections. This could include retraining of MITs in positioning and ensuring that they chose the correct reject reasons on the drop‐down menu. MITs could also be trained on the accurate use of the exposure factors in the DR context.

Furthermore, the anatomical regions that demonstrated significant differences in RRs need special attention with any interventions that are implemented to reduce errors. These anatomical regions include the thorax, shoulder, spine, pelvis/hip and lower limb.

## Conclusion

An overall image RR of 7.86% at RAD 1 and 5.91% at RAD 2 over the 4‐month period that the images were acquired was demonstrated. It was found that there may be multiple factors relating to the selected image rejection reasons and the findings of this study continue to draw attention to the positioning of the axial and appendicular skeleton. Assessment of image RA for each examination type permits training opportunities for MITs to enhance their radiographic skills in specific areas that require optimisation.

## Conflict of Interest

The authors declare no conflict of interest.

## Funding Information

No funding was sought or received for this project.

## Patient Consent Statement

No patient or MIT information was provided with the data collection method, and the spreadsheets were subject to screening by the principal investigator prior to data analysis. No patient or MIT consent was required for this research study.

## Permission to Reproduce Material from Other Sources

The Clinical Director of Radiology at ADHB approved permission for data extraction.

## Data Availability

The data that support the findings of this study are available from the corresponding author upon reasonable request.
